# Foreign Psychological Literature

**Published:** 1857-10-01

**Authors:** 


### Art. V.—FOREIGN PSYCHOLOGICAL LITERATURE.

Since our last *résumé* on this subject, our continental brethren have been pursuing, with their usual zeal and ability, those enquiries which, both in their speculative and practical aspect, form the special objects of our research. The *Annales Medico-Psychologiques*, edited by MM. Baillarger, Cerise, and Moreau, contain much instructive and interesting matter.

M. des Etanges has been engaged for some years in an inquiry into the *Causes of Suicide in France since* 1789. His communication is as yet only introductory; his views not quite developed; yet it is plain that he does not consider suicide inevitably and invariably a mark of insanity or mental aberration. He recognises "disgust of life" as a fruitful source of self-destruction.

"Need we say that the intervention of medicine (strictly so called) is here of no value? We are entering upon an order of facts and ideas, which have no connexion with pathology, and which, consequently, have no relation with, nor anything to hope from, therapeutics. The Materia Medica does not furnish, to our knowledge, any heroic remedy against despair or disgust for life, and it is with astonishment mingled with compassion that we see it advanced gravely by a certain Dr. Retz, that by emetics we may not only cure the spleen, but turn men from all vices. From the material impotence of art, however, in presence of a moral disease, we must not conclude that the physician has only to remain a passive spectator of these incessant strifes, these ardent conflicts of will and instinct, of necessities and passions, in which the activity of society consumes and nourishes itself without ceasing. In this difficult analysis, the physician is the best judge to consult, the best guide to follow. Who better than he to aid the moralist or the legislator! Social torments, and the storms of private life send him sufficient victims to make him acquainted with our miseries and aberrations. Daily witness of the excesses and the miseries of humanity, he knows better than any other by what hideous ulcers the social body is devoured; and thence for him spring the right and the duty to denounce the progress of the evil, and to expose all its deformity."

M. des Etanges expresses a very strong but rather undefined objection to the statistics of suicide as calculated to throw any light upon its causes. He prefers the *weighing* of evidence to its *enumeration*. He proposes hereafter to enter upon an examination of the value of the various causes of suicide under the following division:—

Sect. I.—*Influences exercised by the social condition.*

1. Political events: revolutions, civil wars.
2. Scepticism, incredulity, forms of belief.
3. Imagination: pride, reverie, depression.

4. Fear of dishonour; fear of the police; points of honour; duelling.
5. Domestic trouble, quarrels, threats, and ill-treatment.
6. Love.
7. Wretchedness.
8. Misconduct, drunkenness, debauch.
9. Play, lotteries, speculations, &c.

Sect. II.—*Effects of organic laws.*

1. Spleen, or low spirits.
2. Imitation.
3. Monomania.
4. Hereditary influence.
5. Disease.
6. Mental alienation.

In describing his access to the public documents relating to suicide, he says:—

"Our documents, moreover, are distinguished from most others in this particular, that they serve as an envelope for the instrument used by the suicide, whether poison or mechanical means. In this funereal museum, of course, are not found such arms as are too large to be wrapped up in the papers, as sabres, guns, horse-pistols, &c. Fire-arms, nevertheless, were represented by one little copper cannon, with which a miserable child, aged twelve, had had the cruel *sang-froid* and the fatal address to kill himself. Yet more—full of his resolution, he wished to leave no doubt of the fact, and with a burnt stick he wrote, 'I have blown out my brains on purpose'—*Je me suis brulé la cervelle exprès!*"

M. des Etanges thus concludes:—

"To seize thus upon all the causes of suicide, joining thereto the avowal of the victims themselves—is it not to unveil our moral, physical, and intellectual miseries, and to publish entire the *confession of society?*"

*On Prison Insanity.*—M. Sauze, physician to the prison and asylum of Marseilles, enters upon the question, hitherto much disputed, of the influence which imprisonment, solitary or otherwise, has upon the production or development of insanity. He quotes sundry conflicting opinions, previous to giving the results of his own experience, and his deductions therefrom. M. Ferrus says that Specialists have only in *very rare cases* recognised the existence of mental malady solely due to the despair of detention. M. Lélut, and M. Tardieu, have arrived at conclusions on the whole favourable to the cellular system. M. Lélut establishes:—

"1. That in ordinary societies, there are about two insane individuals in the thousand.

"2. That in all prison life, for reasons drawn from the very nature of this life, the proportion is much more considerable, rising to three, four, five, six, and even fifteen in the prisons on the old plan; but that in the new ones it is not more than two or three in the thousand at most."

These figures prove, says the author, that the modern cellular system is much less unfavourable in its mental influences than the ancient aggregate imprisonment. On the contrary, M. Pietra-Santa, physician of Mazas, investigating the same subject, arrives at conclusions directly opposed to those of M. Lélut:—

"That mental alienation is much more frequent at Mazas than in the public prisons, and,

"That the augmentation of suicides continues to be very considerable. During four years, since the opening of Mazas (where the cellular system is practised) the number has been twelve times as great as in the old 'Force,' or 'Madelonnettes.'"

M. Sauze takes the view of M. Ferrus and M. Lélut, and considers that the population of the cellular prison of Marseilles, approaches, in respect of mental disorder, very nearly to that of the general public. In support of this view, he gives in considerable detail, a report of the cases which have fallen under his own observation, at too great length for quotation; and derives from them the following conclusions:—

"1. The causes of prison insanity are in general independent of the imprisonment, whatever may be the system pursued.

"2. Mental alienation is in general *anterior* to the entrance upon prison life, and even generally to the trial.

"3. When it is developed in prison, it is even then the result of causes sometimes alien to the imprisonment.

"4. The most numerous causes of prison insanity are inherent to the prisoner, and not to the prison.

"5. They consist especially in individual predispositions, such as inheritance, imbecility, idiocy, epilepsy, previous attacks of aberration, or lives of privation or debauch.

"6. There exists the strongest analogy between the insane and a certain class of prisoners composed of men of incomplete organization.

"7. A certain part of the prison population would be better placed in asylums for the insane.

"8. The number of condemnations of the insane is very considerable.

"9. The cases of insanity which manifest themselves in prisons are not due solely to the influence of incarceration; but to various causes connected with general debilitation, and especially the insufficiency of nourishment."

On the 6th, 7th, and 8th clauses, M. Sauze has the following judicious reflections:—

"Besides the direct causes of mental alienation which I have passed in review, there exist in prisons numbers of individuals, whose mental condition, without being that of actual insanity, cannot be considered as one of perfect reason. This intermediate state between reason and folly is the result of an incomplete cerebral organization, and of a vicious education. In such imperfect beings can we admit that there exists a healthy notion of right and wrong? Must we not admit, with Gall, that the free will (*libre arbitre*) in these cases has not the same power and force, and that it is more or less modified or restrained? Is it not evident that these men are driven to crime by the vices of their organization, and that they have a claim to the plea of irresponsibility? For my part, I do not hesitate to answer in the affirmative; and I am sure of having the approbation of all those who think with just reason that the study of organs and their functions is the most solid basis for a sound philosophic doctrine. The analogies which exist between the insane and a certain proportion of our prisoners become still more evident when we consider comparatively the *movement* of the population in our asylums and prisons. It is not uncommon to see one individual sent alternately to prison or to an asylum, according to the varying appreciation of the tribunal. I have often had occasion to observe this remarkable fact. What I say of the civil population, is equally applicable to the military. Our asylum receives soldiers from the army of Algiers and from the Ninth Division. We have very often soldiers, men of defective organization, who are destined all their lives to reside alternately in prison or in the asylum.

"From all this it clearly results that the tribunals daily condemn to punishment insane persons, who should be considered irresponsible. A great number of those condemned for mendicants and vagabonds belong to this category. . . . Let us hope that as these complaints are multiplied, justice will decide at length to appeal more frequently to the light of medicine; and no longer treat with distrust the science which has brought to bear so much precision and vigour upon the appreciation of psychological phenomena."

*On Dreams.*—M. Alfred Maury enters rather elaborately into an investigation of the phenomena of dreaming, with a view to the formation of a theory, which does not seem as yet to be quite completed. His observations are entirely upon his own dreams, and seem to have been carried on for some time in a most systematic manner. It may be questioned, whether his method may not in great measure have influenced or produced the results. He traces in many cases, a strong analogy between the mental manifestations of dreams, and those of childhood or senility. He accounts also for memory and association on physical principles; and also for what is called the spontaneous development of ideas, in a manner which might lead us to suppose that his theory of life and mind was altogether physical or material. Perhaps we misinterpret his intention—but we will quote his own words. Speaking of the apparently spontaneous origin of certain ideas, he says:—

"Ne serait ce pas parceque il se produit en nous, sous l'empire de causes morbides, perturbatrices, ou simplement modificatrices de telles parties de notre organisme, des mouvements qui se répercutent dans le cerveau sur un des millions, des milliards de fibres, de molécules materielles dont il est composé, et là, le mouvement transmis se communique à celles de ces fibres ou molécules que les reflexions ou les préoccupations antérieurs avaient comme laissées douées d'un mouvement vibratoire. Cette explication rend compte également de la mémoire spontanée, phénomène si etroitement lié à celui de le génération spontanée des idées."

And further on he observes :—

"These spontaneous vibrations are certainly placed under the dependence of the different regions of the organism, of which they are the echo."

After the enumeration of many facts connected with the frequent hallucinations in the half-waking state, and other phenomena of perfect dreaming, he continues :—

"We may say, in presence of these facts, that man is an automaton, WILL occasionally influencing or winding up the spring—Habit being the balance. This automaton continues to act when the will is absent, so long as the spring unwinds itself. Once the clock wound up (*l'horloge montée*) the works continue to move, a little influenced, however, by exterior causes and internal modifications attendant upon their composition and nature. In the best made intellectual clocks, that is to say, the strongest and soundest intellects, the intermission of the action of the will takes place at extremely short intervals; but the more enfeebled the intelligence, the less active is the will, and the more constantly the machine is left to obey the law of automatism which is proper to it (*qui lui est propre*). It is but justice to M. Maury, however, to state that in the close of his paper he does verbally recognise an immaterial existence in the words, 'cet univers invisible et immatérielle qu'on appelle l'intelligence.'"

*Observations on General Paralysis*, by M. Baillarger.—According to the statistics of MM. Aubanel and Thore, there died in the Bicêtre, in 1839, 164 insane patients, of whom 125 were affected with general paralysis—three-fourths of the whole. In the hospital of the Senavra at Milan, devoted to the same class of patients as the Bicêtre, there died in 1853, 60 insane persons, of whom one only was affected with general paralysis. On the rarity of this affection in Italy, Esquirol speaks very decidedly; and M. Guislain considers it as almost entirely absent. "This," says he, "is the more extraordinary, as this affection has been often considered as a result of inflammation, and as Italy is especially a country where inflammatory affections are frequent and intense."

M. Baillarger, whilst recognising the comparative infrequency of general paralysis in Italy, considers that it is more frequent than appears from the above statements. The discrepancy seems to arise from many of these cases having been described and classified as *meningite lente*, a very frequent affection, treated by a more or less energetic antiphlogistic treatment. M. Baillarger gives the details of a considerable number of cases which were thus classified, and which were certainly undistinguishable from what is now generally recognised as general paralysis; characterized by embarrassed speech and gait, progressive paralysis of the limbs, and ambitious delirium. The most frequent morbid change found was thickening or adhesion of the membranes.

*On Simulated Insanity.*—There are numerous cases related in the recent journals of medico-legal investigations into the mental condition of criminals, all containing more or less matter of interest. We select one of simulated insanity, investigated by M. Morel, as especially instructive, from the careful observation of facts, and the highly philosophic method of induction from these. The *instruction* related that Pierre Derozier, aged forty-one, hawker, without domicile, was accused of twelve robberies of churches. On the 26th of January he answered with perfect clearness to the "juge de paix" of Gournay, and acknowledged himself guilty, entering into the most precise details. He spoke of an accomplice by the name of Chapoteau, who has not been found. He was still coherent on the 1st and 5th of February. On the 12th of March, he refused to reply to interrogation, and preserved absolute mutism, also on the 4th of April. The 13th of May he made incoherent and irrelevant answers, and has since then given way to many insane acts and some violence, and occasionally said that he was mad. M. Caron, of Neufchatel, had examined him and reported him insane.

M. Morel, having taken the usual oaths, was introduced to the prisoner, whose personal appearance he describes minutely, and also his actions. He was restless and incoherent, perpetually moving about and turning round on his axis. The name of Chapoteau continually occurred in his discourse. He had robbed him of thirty-five millions, and ought to be shot. He refused to eat, professing to fear poison. In the night he was tranquil. "The existence of Derozier in the daytime is that of some automatic and extravagant insane persons. He sits in the corner, balances himself from right to left, or backwards and forwards; he picks up bits of straw and feathers; his eyes are half shut, and perpetually winking." The physical condition was in all respects normal; no sign of general paralysis. Interrogated as to his age, he replied "245 francs, 35 centimes, 124 carriages, &c., &c." To the same question, more distinctly asked, he replied, "5 metres, 75 centimetres."

*Q.* Have you been long deranged?—*A.* Cats, always cats. Yes, I am not mad.

It is unnecessary to go through all the details of the interrogatory. All his answers were determinedly incoherent, having no relation whatever to the question.

M. Morel thus reasons upon the phenomena :—

"I do not hesitate to say, that the answers of Derozier are not those of an insane person. In their extreme aberrations, in their most furious delirium, *madmen do not confound what it is impossible for the most extravagant logic to confound.* There is no madman who loses the idea of *cause*, of *substance*, of *existence.* I will explain by examples. If we ask an insane person his age, he may answer six millions of years or six months—he may say that he is no age, because he is dead; but the most incoherent madman will never reply 245 francs, 35 centimes. If asked concerning his parentage, he will say that he is the son of the king, the emperor, or of God; but he will never make a reply which could have no reference to causation, to substance, or existence. Once more, the most incoherent will not confound the idea of time with that of distance, &c.

"But leaving abstract psychology, let us inquire to what class of the insane does Derozier belong, if he be insane."

M. Morel successively excluded from consideration general paralysis, mania, dementia, melancholy, and cleptomania, by an elaborate argument, and considering that Derozier simulates insanity, and simulates it unskilfully, he reported to that effect to the judge. A second examination, with etherisation, led to the same conclusion. After a very long consultation, the jury condemned him to the "travaux forcés." Immediately after his condemnation, his insanity ceased, and he acknowledged the fact of simulation, his reasons, and the difficulty he had experienced in sustaining his part, in language which evinced a more than ordinary amount of intelligence. His concluding words were rather remarkable. On being urged to attempt by good conduct to merit some commutation of sentence, he replied with a melancholy shake of the head, "Once entered on an evil course, it is scarcely possible to leave it. I am forty-two years old; it is too late. I retire now from the world; I enter the cloister; my *rôle* is played out."

*On Delirium Tremens.*—M. Pinel, jun., has published recently a short treatise on delirium tremens, in which he proposes a new method of treatment, that by bathing, almost without other aids. He enters at some length into an examination of the symptoms, particularly as to their diagnostic value, and traces skilfully the points of distinction between this affection and others with which it may possibly be confounded in the earlier stages of its apparition. These are meningitis, meningo-cephalitis, encephalitis, typhoid fever, nervous delirium, acute and maniacal delirium, other forms of toxic delirium, the delirium that succeeds epilepsy, and dementia with or without general paralysis. He considers that it is often very difficult to form a correct and ready diagnosis between this latter disease and delirium tremens, especially as the one is often complicated by the other. There are, however, certain distinctive marks which must be borne in mind. In mania complicated with general paralysis, the tongue is ordinarily not foul as in delirium tremens; the appetite is good; the breath is not strong and alcoholic; the skin is dry and harsh; the loss of sleep is rarely constant; the physiognomy is happy in expression; the delirium generally turns upon riches or grandeur; the hallucinations are as a rule essentially distinct from those of oinomania.

After reviewing the methods of treatment which are or have been hitherto employed, M. Pinel gives the preference to opiates, but complains of frequent want of success even by this method. He then says:—

"Prolonged hot baths, with continual aspersions of cold water to the head, appear to be the best means of cure for delirium tremens—with or without opiates. For fifteen years we gave these conjoined with the hot baths; now, we have almost renounced them, finding that the baths alone are sufficient to effect a favourable result in one, two, or three days at the most, without the least risk to the patient."

The special apparatus is described at some length by which the hot bath is kept up at the same temperature, and the cold constantly applied to the head. M. Pinel states:—

"The duration of the bath varies from one to five, ten, fifteen, or even twenty hours, according to the intensity of the affection. It is not prudent to continue it longer—it is better to renew it on the morrow. After their bath, we prescribe a hot water drink frequently, so as to keep up perspiration. During the bath also, lemonade or some diluent is taken frequently."

Some cases are given in illustration of this method of treatment, from which it would appear to be eminently successful. Except in hospitals, however, it would appear to be difficult of application. M. Pinel enters slightly but judiciously upon the general bearing of the question of drinking upon social and national relations; and also discusses the propriety of the isolation of those who have had frequent attacks and relapses into the vice. The social and legislative difficulties attendant upon this point appear, however, at present to be almost insuperable.

*Case of Poisoning and Acute Mania from the use of a Cosmetic.*—M. Moreau relates a case of poisoning and mania resulting from the use of a cosmetic containing lead and other poisons, which is sufficiently interesting to be quoted at length.

"Valleau, æt. 29, hairdresser, entered the Bicêtre on the 9th of

June, 1855; he was examined, for the first time carefully, on the 11th. He was in a state of profound stupor, from which nothing would rouse him—the eyes were immoveable. One remarkable phenomenon struck us—the hairs of the pubis and of the chest were perfectly white—those of the head were, some white, some rather red, and some with a blackish tint. Each of the cheeks and part of the neck had greyish-yellow patches upon them, like jaundice. Similar spots were found on the left thigh.

"There was no fever; the tongue was a little loaded, and there was constipation. M. Moreau prescribed a bottle of seidlitz water, and two issues to the neck.

"On the 12th he did not answer to any questions, but seemed to be uneasy, and to make efforts to speak—he frequently raised himself in bed.

"On the 13th he was in a more satisfactory condition—he replied to our questions, but seemed to have lost his memory, and gave a very imperfect account of what had happened to him—he knew that he was a hairdresser, but not where he lived—he remembered having invented a pomade for the hair, and having used it himself, but could not remember the composition.

"On the 14th he was completely recovered from the stupor, and was much distressed to find himself at the Bicêtre—he gave with much precision the details which we required; for some years back, his hair had been turning white, and to remedy it he had composed a pomade, of which the elements were very active—viz.,

| | |
|---|---|
| Litharge . . . . . | 400 parts, |
| Quick Lime . . . . | 200 „ |
| Prussiate of Potash . . | 50 „ |
| Nitrate of Silver . . . | 20 „ |

Fifteen days before his entry, he had commenced using this compound, and in three days had used above a pound and a half! The hair became partially black, but accidents began to occur; he had violent colics and headache. By his own account, his intelligence remained clear, but his employer from the first observed a change of character; he had become sad, and performed his duties with less skill than before; he continued to work still. But on the 9th of June, although twelve days had elapsed since he discontinued the use of the pomade, he became ill, and remembered nothing from that time till his awaking to consciousness in the Bicêtre on the 14th. Information given by his employer supplied the gap. Delirium set in suddenly, and the patient became violent; he threw on every side his instruments, and tore his papers; he believed that he was pursued by his enemies, who wished to poison him. In this condition his employer sent him to the Bicêtre, where stupor succeeded to the previous excitement.

"On the 15th, a little weight on the head, and some gripings were all the remains of the illness. On the 19th he was quite well, and was discharged."

M. Moreau naturally attributes this affection to the head; and considers, in opposition to the opinion of some toxicologists, that it was through the skin that the effects were produced. He concludes with the following remarks :—

"Whatever may have been the mode of introduction of the poison, there are facts in connexion with the subject of the highest importance to those who use habitually such cosmetics. It is well known how extensively this practice prevails amongst dramatic artists, and also amongst females of a certain class. All physicians, those especially who have any professional knowledge of actors and actresses, know how subject they are to nervous affections of all kinds, from the slightest to the most formidable. Instead of constantly referring these affections to the baneful influence of moral impressions, as it is so much the custom to do, would it not be more rational and more scientific (although perhaps less poetical) to suspect simply lead poisoning? This question we only suggest, leaving it to others to decide."

*Treatment of Epilepsy and Chorea.*—M. Trousseau, of the Hôtel Dieu, has the following observations on the treatment of epilepsy by belladonna :—

"I have always a number of patients in Paris and the departments under treatment. In some the belladonna fails entirely, in others it has brought some relief. This is my mode of procedure: I give a pill containing one centigramme of the extract and an equal quantity of the powder of belladonna during the first month—by preference in the evening; partly because of the inconveniences attendant upon this remedy at first, and partly because epilepsy is most frequently nocturnal. After a month I give two such pills at once, for an equal time—and then three, providing it be well tolerated—if not the dose is only increased once in sixty days. A register is kept of every attack—if at the end of six or nine months, or a year, their frequency is decreased, I press the remedy, for I know that the disease is yielding. By proceeding thus, you will moderate the frequency of epileptic attacks in many cases—in many others, however, you will obtain little or no benefit. In twelve years I have thus treated 150 patients, and have cured 20. But will not even they relapse?"

In chorea M. Trousseau has introduced the use of strychnine with great advantage; but as to produce its proper effect it has to be given to such an extent as to produce its specific physiological results, it requires constant supervision and watching. It is given in small but continually augmented doses, and even to young children; the reports of the cases show a slight but marked advantage in this over the previous methods of treatment in the time required for cure.

Dr. Legrand du Saulle, in the *Annales Medico-Psychologiques* for April, 1857, after noticing M. Herpin's treatment of epilepsy by the oxide of zinc, has the following observations :—

"It may be remembered that M. Champouillon has related many cases of epilepsy in soldiers suffering from anasarca, which cases he attributes to the pressure of the serosity upon the nervous centres—a view confirmed by the fact that the anasarca and the epilepsy have simultaneously decreased under the influence of hydragogues and drastic purgatives.

"This idea of the intervention of pressure in certain forms of epilepsy seems to have been the foundation of the curative method introduced by M. Hiard. According to this authority, epilepsy results from *the interruption of the electro-vital fluid.* This interruption is produced by a transitory congestion of that part of the spinal ganglion included in the occipital foramen. The therapeutical indication deducible from this purely ideal view, is to combat the intermolecular stasis by means of bleeding; but M. Hiard prefers purgatives and cutaneous revulsives.

"During the first month of treatment, two purgatives of castor oil are given every week; afterwards reduced to one, which is continued until convalescence. At the same time he employs slight vesicants alternately to the legs, and liniments of camphorated spirit to the body generally. M. Hiard never bleeds nor uses diuretics, because he says the oil has always succeeded, at least in recent cases."

*Idiocy and Cretinism.—Contributions concerning Idiocy.* —The first of these contributions is an elaborate and able paper, by Dr. Kern, of Leipzig, from the *Allgemeine Zeitschrift,* which we present abridged.

"A glance at the strivings of the present time shows how public, national, and private institutions have proposed for themselves the aim of spreading a truly moral and religious education; inasmuch as the conviction has arisen and grown into a lively and active principle, that only an educated can be a happy and contented people. In order to begin the elevation and ennobling of the race at the source, there is at present scarcely to be found a village which does not contain one or more institutions for the care of children; and freely does the government or the public contribute to found new ones, and to place the old on a more effective footing.

"This care is not only extended to the general, but also to the special, wants of the community; so that those classes of our suffering fellow-creatures who, without special means and institutions for education, must remain neglected, have attracted active sympathy. Scarcely any city of the civilized world is at the present time without institutions for the care of the deaf and dumb and the blind; and in recent times the imbecile and idiots, hitherto supposed incapable of improvement, have been made the objects of a like care.

"It would be useless to inquire from whom the first proposal emanated for the education of these unfortunate classes; for whilst Director Saegart, of Berlin, in 1846, believed that he had demonstrated the curability of idiocy and imbecility, the endeavours of Seguin in Paris were directed to the same end; and even before Dr. Guggenbuhl opened his institution on the Abendberg, in Switzerland, Voisin in Paris had experienced very satisfactory results in the same department in 1836; even Voisin himself had predecessors. It is in this, as in so many branches of science and art—the same idea emanates synchronously, and is followed out in various places, indicating that it is a necessity of the time.

Amongst those supported by government are that of Hubertsberg, in Saxony, those of Mariaberg and Winterbach, in Wurtemburg, and oné in Sardinia. None else are known to me which are devoted to the care of idiots and imbeciles exclusively; but private institutions for the same purpose have sprung up, mushroom-like, everywhere. Unqualified persons have sought to take possession of this ground; yet such institutions as have been erected from mercenary motives have for the most part fallen to the ground, or are undergoing such a decline. Others interested in such projects have lost faith from these failures, and have conjectured that in a little time no one will entrust the afflicted child to such an institution. From my many years' work in this department, I am convinced that the endeavour to educate this unfortunate class will ever meet with more and more favour, provided that, on the one hand, too much is not *expected*, and, on the other, too much is not claimed as an aim. I am the more confirmed in this view by the results of a journey, taken for purposes of inquiry, which will be here detailed."

Dr. Kern proceeds first to speak of the meaning and relations of imbecility, cretinism, and idiocy. Defining imbecility to signify that condition of mind in which the "inner life-sphere" is not characterized by the capabilities of the sound mind, he inquires whether a normal psychical development has preceded this state. The argument concerning the propriety of the term "congenital" is long and elaborate. He states that we can with no more propriety speak of a truly congenital imbecility than of a truly congenital preparedness for all intellectual manifestation; that there is no doubt that the mind is not originally what we infer from the contemplation of educated men, a something prepared for this education from the beginning; but a result, a product of surrounding nature, animate and inanimate, of internal development, of external communion with previously existing intelligence, of fate, its author and guide. He considers, also, that the capacity of development, even to the highest grades of intellectual eminence, is an original right and possession of every viable child; that errors in intellectual as in moral training may enter as false elements in the mental structure, but that the *mind* cannot be originally diseased.

The question then arises when and whence the disorder originates which we speak of as congenital, a question truly, which in the concrete is most difficult of solution. Dr. Kern then sketches, at considerable length, the contest of the child's nature with the surrounding influences; and the various physiological changes which accompany the somatic development; also the varied circumstances as to care and hygiene, which in some cases assist, in others counteract the morbid influences—diet, poverty, and neglect; as contrasted with cleanliness, vigilance, and well-directed treatment at critical periods. It is well illustrated by the consideration of the process of dentition, in which the functions generally are roused to a state of great activity; when there is a tendency to hyperæmia, especially in very vascular organs, such as the brain—aggravated perchance by pain, and by casual circumstances—passing on to inflammation, and formation of inflammatory products not perfectly absorbed. In one case, well-directed care may constantly meet and counteract these evil tendencies, and the child will pass over the critical period, with more or less safety, in the other, the results will manifest themselves in imperfect or morbid somatic and psychical development, which about the second or third year will begin more openly to show themselves.

"The skin is pale and withered, the muscular system is flabby, the cellular tissue lax; disproportions are manifested in the head, or extremities. Then we observe the long, or perhaps unnaturally wide face; the wrinkled forehead; the spiritless, often inflamed or squinting eye; the large, red, projecting ear, often with an offensive discharge; the thick lips, which yet can scarcely cover the irregular large teeth; the contracted or distorted thorax; the tumid belly. The child knows no refreshing sleep; it lies disturbed, or awakes with a cry; diarrhœa and obstinate constipation alternate; and the original muscular twitchings have developed into true cramp or convulsion. Unable to support the weight of the body, the child does not make the same exertions in locomotion that healthy children of the same age make. The phenomena of the external world pass without trace over him; no ray of joy enlivens the dull eye, no smile irradiates the fixed, pain-expressing countenance; no attempt at speech or articulate sound is made, or only a few words imperfectly learned, as necessary to the supply of the physical wants. Viewing this as a whole, we have a picture of the perfect scrofulous or rachitic diathesis, which through all relations of life will ever more and more become prominent. The development of this diathesis to the highest grades depends upon the intensity of the morbid processes and upon the external circumstances attendant upon it. The child which enjoys judicious care and treatment may survive happily enough the process, even although the tendency has been inherited strongly from generation to generation. On the other hand, the child, born and brought up amid misery and poverty, will sink into the above described condition, which, if placed in more favourable circumstances, would scarcely have manifested any, or only the mildest symptoms."

Dr. Kern then proceeds further to illustrate the position that cretinism, whether sporadic or endemic, is a highly developed form of scrofulous or rachitic tendency; and that in the districts where it is endemic, the gradations are insensible from the mildest of those affections, to that which is truly reckoned cretinism. He shows that the prophylactic and hygienic resources which are notoriously useful in the one class of affections, exercise the same favourable influence upon the other, and controverts with some asperity Dr. Guggenbuhl's theory as to the distinctness of these affections; first on general argumentative grounds, and next with regard to Dr. G.'s peculiar and individual notions.

"Further, Dr. Guggenbuhl draws a distinction between common idiocy or imbecility and cretinism, on the ground that the latter, once waked from the brain-slumber, before all things is accustomed to recognise the existence of a Deity, even before he comes to an understanding of surrounding objects—for instance, the table before him. Such are ideas to communicate to old women, but not to make scientifically available."*

Dr. Kern recognises some other causes of defective intelligence —pressure on the head, premature birth, difficult labour, certain forms of general disease, and excitements of particular parts of the peripheral nervous system, thymic asthma, hooping-cough, &c. And as the causes are various in the nature and intensity, so the results produced are so various as to elude classification; for whilst some are so slight as to differ but little from the healthy manifestations, there is every grade from this to that condition where there is scarcely a spark of soul to distinguish him from the beast. He afterwards notices how frequently the absence of all anatomical lesion is remarked in the most confirmed cases, both in general and microscopic examination; and then passes on to the different systems of recognition and treatment by their different men, or schools, SEGUIN in Paris, GUGGENBUHL in the Abendberg, and SAEGERT in Berlin.

"The fundamental views of these three men certainly differ materially. Dr. Guggenbuhl considers cretinism as an independent form of disease, expressing itself by bodily and mental crippling (*verkrüppelung*), congenital, or developed in infancy, up to the seventh year. His conviction that even children affected with, or having a tendency to, cretinism, may be normally developed if removed from the valleys to the mountains, has induced him to build his institution on the Abendberg. To cretinism Dr. Guggenbuhl opposes congenital imbecility and idiocy, as incurable."

* From Dr. Guggenbuhl's letter concerning the Abendberg:—"One day, as the setting sun gilded gloriously the evening sky, the noble spectacle attracted at once the attention of all the children of the establishment. Joy, astonishment, rapture, and wonder seized them all; and even F., a boy who hitherto had been shy and unsociable to friend or foe, indifferent to pleasure or pain, and *dumb*, called suddenly out, 'The sun!' The ice-rind of the soul was broken; he even spoke further to his companions, though his powers of conception were still so weak, that he could not distinguish between the fingers of his own hand."—It will be borne in mind that in all Dr. Kern's strictures upon Dr. Guggenbuhl, and the system of which he is the exponent, *we merely quote.*

"Seguin founds his hope of happy results in the treatment of imbecility on the fact, that even the flea may be educated or taught.

"Director Saegert lastly considers imbecility as a *condition*, not a *disease* of the mind; conditioned by a pause at a low grade of development, and proposes to attempt to take up the mind at that grade, and lead it forward intellectually *towards* if not *to* its normal condition."

No account is given by Dr. Kern of Seguin's method, and only a brief allusion is made to that of Saegert, further than to imply that his method of treatment is purely intellectual, and that the results are still unpublished. An extended notice of Dr. Guggenbuhl succeeds:—

"Dr. G. adopted another idea—he saw a cretin lie praying before a cross, and the thought struck him that he must be his saviour. He put his hand powerfully to the work, and diligently extended the rumour of his call throughout the world. Yet, indefatigable alike amongst the children and at his writing-desk, he appears neither to have suggested any new scientific aspect of these disorders, nor any new method of treatment.

"In my inaugural dissertation 'De Fatuitate, &c.,' I have expressed the opinion that I could not place implicit confidence in all Dr. G.'s published details; but it was long my wish to see him actually at work. Accordingly, on August 30, 1853, I visited the Abendberg, and was told, after an hour's delay, that Dr. G. was ill. After another long delay, I found the children assembled in a large room, which served for dwelling-place and school-room; it was provided with an organ, a Chinese drum, several forms of orthopedic apparatus, a running machine, and pictures of all kinds. The children were playing briskly; I took two by the hand, and said to the female attendant, 'These are the pikes in the carp-pond,' meaning the elements of life and health. She replied, 'These boys were very bad, they have cost us much trouble.' With this she looked so suspiciously at me, that I could scarcely conceal my displeasure, for I considered the boys healthy both in mind and body. Seeking to converse with the other children, I was prevented by various means—thus, if I spoke German, they were French children—if French, they were English; and getting a little German girl before me, I was told she never spoke when strangers were present!

"A boy showed me a copy-book, in which were sentences written in German and French; yet I found he could not tell the month, nor the day of the week, nor his age, nor birthday. For the rest, I found precisely the same forms of the affection as we have in Germany—brain affections, and imbecility resulting therefrom, in its higher or lower forms;—and rachitic and scrofulous diseases, mind and body thereby deteriorated.

"It was noon, and I left the institution, mounting the Abendberg. I expected to see the children come out to enjoy the glorious weather, but I looked in vain; only one was visible, swinging round and round a tree. The next day was bitterly cold; shivering, I visited again the institution, and found the children also blue and almost stiff with cold.

This day, accompanied by Dr. G. himself, I witnessed the process of education. About twenty children were taught by two female teachers—one for geography the other religion. The first sat before a map, pointing here and there with a rod—'What is that?'—'That is England,' rung out the answer of the children: 'What is this?'—'That is Ireland, London, Dublin,' &c. The second teacher had pictures representing Bible-history, which she explained, and repeated passages of Scripture concerning."

Dr. Kern was very much dissatisfied with his visit, and gives details of several very important modifications to be made in the published statements concerning this institution. He disapproves strongly of speaking of the *cure* of idiocy, when such cure consists only in the ability to write out sentences from copies. Then follow brief notices of Dr. Erlenmeyer's establishment in Bendorf, that of Dr. Zimmer at Mariaberg, and that at Winterbach, under the care of Dr. Müller. Of the care exercised over the children in these places, Dr. Kern speaks most highly, but seems to imply that he has not met with any *cures* in well-established cases:—

"Indeed, of *cures* of imbecility I believe we cannot find one credible case; for the accounts given by Dr. Saegert in the second part of his work are as imperfect as would be the account of a taliacotian operation at the moment of its completion; they go no further than to say of the children—'They are in *course of* full development.' This was in 1845, and if we inquire into the after progress, we have no data."

If idiocy or imbecility dependent on organic mischief in the nervous centres be insusceptible of cure, an important inquiry suggests itself, as to whether all who appear imbecile or idiotic are thus, or in some degree organically diseased. Dr. Kern answers this in the negative. Development of mind is arrested or retarded in many cases by accidental circumstances, by lack of care or attention, by many attendants upon poverty and misery. Mind again is worn out by over-forcing early, as in infant prodigies. In neither of these classes can *disease* be correctly predicated. Hereditary weakness of frame in a child may lead to extremely slow development, merely from inertia; and may strongly simulate imbecility. All these and many allied cases are favourable for an attempt at cure. After enumerating certain diseases which often are attended by mental imbecility, Dr. Kern makes some interesting observations upon the effect of the organs of sense upon the intelligence:—

"The organs of sense are the media through which those parts of the brain concerned in mental life receive their stimulus. If these be imperfect, so will the psychical development be imperfect, as we see in the deaf and blind, and the bodily organs themselves will be backward in development, as a consequence of their inactivity; as, on the other hand, the use of any of the senses causes a fuller development of its corresponding nerve. Cases are not rare where, for instance, scrofulous children, from their earliest infancy, are so affected with ophthalmia or otitis, that they receive nothing but painful impressions from the external world. Here we should expect an abnormal condition of mind to result, without supposing that the brain itself must necessarily be affected."

One of the most important practical points dwelt upon by Dr. Kern is, the personal care of these afflicted children, as to diet, cleanliness, &c. He speaks of alternating diarrhœa and constipation, not so much as the result of even functional affections of the bowels, as arising from neglect of systematic evacuations, whereby the bowels become loaded, till an effort of nature compels attention. The same is the case with the bladder, whence most frequently results the wetting of the bed. Long confinement to bed he considers a very frequent cause of onanism, from mere lack of employment for the hands. The cure of these evils, he thinks, is to be sought in perpetual watchfulness, night and day, and in systematic periodic relief to the bowels and bladder—in the one case, bad habits will be broken, and in the other, forgotten.

In instruction, Dr. Kern has the greatest faith in familiarizing the children, not with pictures and diagrams, but with the use and nature of common things, such as everywhere surround them; so that if the mental affection be really incurable, the body may be trained to some degree of order and utility.

The paper concludes with a forcible representation of how much may really be accomplished in this department, and a strong appeal to those interested in the subject to persevere.

Dr. Müller himself gives an account of his institution at Winterbach, in his sixth annual report. Prominent features in his system are cold-water baths, "Swedish gymnastics," and the administration of sulphur, in minute doses. The children are divided into three classes, each having a separate teacher. The first includes about 20 children, from whom we are told several are selected every year as adapted for certain callings in life. The second class contains about 17, and the third the remainder. The entire number is 37 boys, and 29 girls. The deaf and dumb are not now admitted, but sent to a separate establishment. The diseases chiefly prevalent amongst them are typhus fever, hooping-cough, dysentery, and tuberculous affections.

The peculiar part of Dr. Müller's report is that which treats of the origin of cretinism and allied affections. He attributes it to *marsh-miasma*, which when acting "*intensively*," produces intermittents; but acting slowly and gradually as a poison, produces cretinism, goître, and the deaf and dumb condition. As these affections, however, occur endemically, when the cited cause does not exist the author had recourse to a theory which, Dr. Erlenmeyer very justly remarks, removes all such difficulties—viz., that the miasm can be conveyed any distance as mist. Besides this, there are secondary causes which are recognised as having some influence, as the drinking of brandy, bad diet, want of cleanliness, moral and mental neglect, damp dark dwellings, hereditary affections, &c. &c. His prophylactic measures are in accordance with the above theory.

Herr Gläsche, the principal teacher of the institution for imbecile children at Hubertsberg, gives some account of the method in use, and the results. It was opened in 1846, but placed on a more definite and extended footing in 1850. Dr. Erlenmeyer, commenting upon the *method*, observes that in this as in most others, there is one prominent defect—viz., that of attempting the instruction of the children either as if they were of sound mind, or as if they were deaf and dumb; in the latter case using too much pantomime and pictorial illustration, and in some measure neglecting the very important road to the intellect through the ear.

Since the opening of the establishment, 45 children have been received—30 remain under care; 2 have died; 6 have been removed either to other similar institutions or taken home; 7 have been discharged as competent to perform certain functions in public life.

Dr. Erlenmeyer, one of the accomplished and laborious editors of the *Correspondenz Blatt*, gives an account in that journal of the recent opening of the "School for Idiots" at the Hague. As at his visit the establishment had only been open three months, much progress was not to be expected; but there were twelve boys and ten girls, in five classes, the upper three having each a male, and the other two each a female teacher. He was much and agreeably surprised with the order and system already introduced, and with the exactness and unity of energy with which the plans were carried out. He speaks with great praise of the *oral* instruction, and mentions incidently how frequently it is the case that children of the class for whom these institutions are intended are susceptible in no common degree to the influence of sounds, rhythmical or musical—an important hint as to education. Of details it was still too early to speak. The establishment is intended for *curable* cases—those suffering from actual insanity, from epilepsy, or other incurable affections, are not admitted. Young children are preferred; but they are received up to the age of twenty-five years if any hope of amendment is perceived. The treatment is somatic and mental—gymnastics, music, singing, speaking, reading, writing, and reckoning; lastly, simple forms of manual labour. When they are sufficiently advanced to be fit for an ordinary school, the mission of this institution is complete. They are discharged under these conditions: when they are twenty-eight years old, or when they have been five years without making progress; when the state of health is such as to make their further continuance there either hurtful to themselves or to the other children ; finally, when all hope of improvement is given up.

Dr. Erlenmeyer, approving most highly of this school, suggests two points especially where improvement is desirable. The first is as to the situation of the building; it ought not to be in a town, but in the country ; the countless canals, the evaporation, the scarcity of pure drinking water, render the town inexpedient. The neighbourhood of the sea, and a rural district, are very important. The second point is the necessity of a resident superintendent physician in all such establishments. This is enforced by many strong arguments.

In an interesting account of the asylum for idiots at Mariaberg, by Dr. Zimmer, there are the following remarks:—

"All the children in whom imbecility or stupidity is prominent show also bodily defect or misproportion. In the most favourable cases there is generally some smallness of the body, looseness of conformation of the limbs, shuffling gait, disproportion of head (either too small or too large), low forehead, flattened occiput, dull eye, open slavering mouth, &c. A natural classification of the inhabitants of this institution (about seventy in number) would be as follows:—

A. Entire Cretins.

I. *Motionless.*

Body small—brain atrophied—extremities useless or palsied, sensation defective and dull—dumbness—difficult digestion. Mere vegetation; defective consciousness—lethargy and sleeplessness—catalepsy and epilepsy.

II. *Locomotive* (automatic).

Brain atrophy or hydrocephalus—automatic motions—dulness of senses. Animal (and musical) sounds. Greediness, instinct. Simplest form of comprehension—caprice—epilepsy.

III. *Restless.*

Brain atrophy or hydrocephalus—constant unrest—stammering or dumbness—unnatural appetite—sexual excitability—self-consciousness—passion, destructiveness. Ear for music—tendency to mania.

B. Half Cretins.

Some fitness for improvement—some mechanical and spiritual tendencies—some powers of thought and speech.

These again are divided into—

IV. The torpid or clumsy form, and

V. The agile, or useful (brauchbare).

This latter approaching in many particulars to a healthy form of development.

"In order to form a *prognosis*, it is above all necessary to remark the bodily condition. When there is defect of brain, there is scarcely ever much improvement to be looked for. A criterion of this is afforded by measurement of the head. Dr. Erlenmeyer first pointed out that where the sum of the diameters (in length, breadth, and depth) of the head amounted to less than the circumference, there was defect of the brain. The complication with well defined insanity (geistesverwirrung) is very unfavourable as to prognosis. So also is epilepsy. The more the mass of the head preserves its relation to the mass of the body—the more the anterior part of the brain is developed—the younger the child—by so much the more a favourable result is to be anticipated. In such cases, paralysis of the extremities, or displacement of the bones of the skull or face, is not of very serious import."

The arrangements of this institution, both as to personal care and supervision, hygiene, therapeutics, and instruction, seem to be most excellent; but our limits forbid further details.

Not remotely connected with this subject we remark some interesting statistics on the *inheritance* of cretinism and deaf-dumbness, by Dr. Meyer-Ahrens, of Zurich, in the *Correspondenz Blatt* for the 28th of February last. We will arrange his results in a tabular form. Out of 765 cases in which cretinism in some form was observed in families, the parents, one or both, were affected as follows:—

The first column gives the nature of the affection; the second the number of cases in which the father only was affected; the third the same with reference to the mother alone; and the fourth the number where both were so.

| | F. | M. | Both. |
|---|---|---|---|
| Imbecile | 1 | 4 | 1 |
| Simple or weak | 7 | 6 | 10 |
| Religious mania | 1 | 0 | 0 |
| Low form of cretinism | 0 | 3 | 6 |
| Melancholy | 0 | 3 | 0 |
| Hypochondria | 1 | 0 | 0 |
| Impediment of speech | 0 | 2 | 0 |
| Deafness | 2 | 0 | 0 |
| Scrofula | 3 | 1 | 3 |
| Paralysis | 0 | 1 | 0 |
| Sickly | 0 | 3 | 0 |
| Epileptic | 1 | 0 | 0 |
| Deformed | 2 | 2 | 2 |
| Dwarfish | 0 | 0 | 0 |
| Drunkards | 12 | 2 | 3 |
| Healthy and intelligent | 0 | 2 | 179 |

In 35 of the above cases other brothers and sisters were cretinous.

In eleven cases of deaf-dumbness, one had a feeble mother; six had both parents cripples; one had a father and another a mother goitrous; two only had both parents healthy.

It appears from other tables, that the line of inheritance is from father to daughter, and from mother to son, very much more frequently than conversely. Thus, in one series of cases, the disease was inherited from the father by the son 7 times, and by the daughter 17 times; whilst it descended from the mother upon the son 17 times, and upon the daughter only 6. In another series the proportions were—father to son 6 times, to daughter 13—mother to son 12 times, to daughter 4.

There are several papers in the *Zeitschrift* which well merit an abstract, did space permit; we must be content to enumerate the most important. Dr. Nitzsch gives an able and interesting account of the state of psychiatry past and present in Egypt, from which it appears that although much has been done of late years, there remain many relics of ancient barbarism, from the times when maniacs were viewed as little more than wild beasts. Dr. Esmarck and Dr. W. Jessen, relate some cases illustrative (though according to their own views not conclusively so) of the connexion in many cases between syphilis and insanity. Dr. Brosius has two interesting papers, one upon the "Mechanism of Sensation," and the other upon the "Speech of the Insane" viewed as a diagnostic sign. Perhaps at some future time we may be able to give an analysis of these able contributions.

